# Brain photobiomodulation therapy for acute sport-related concussion: a randomized, single-blinded, pilot clinical trial (RECOVERY study)

**DOI:** 10.3389/fneur.2026.1894623

**Published:** 2026-09-22

**Authors:** Jean-François Chermann, Philippe Malafosse, Guillaume Champleboux, Julie Bisiaux, Sara Guillemin, Meriem Benmerad, Andréanne Varet, Patrice Cristofini, Guillaume Blivet, Jacques Touchon

**Affiliations:** 1 Georges Pompidou European Hospital, Paris, France; 2 Private Practitioner, Montpellier, France; 3 REGEnLIFE, Paris, France; 4 RCTs, Lyon, France; 5 University of Montpellier, Montpellier, France; 6 JT Conseils, Montpellier, France

**Keywords:** brain, clinical trial, concussion, mTBI, neuromodulation, neurotech, photobiomodulation, sport

## Abstract

**Background:**

The current standard of care for concussion relies on symptom management; there are almost no disease-modifying treatments. The RECOVERY study investigated the safety of RGn550 (REGEnLIFE), a novel neurostimulation device combining brain photobiomodulation with a static magnetic field, in sportspeople with acute concussion and described concussion-related parameters in patients receiving this therapy.

**Methods:**

This prospective, randomized (central randomization, 1:1 ratio), single-blind, monocentric, pilot study compared two pulse frequencies (5 Hz and 10 Hz). Adult sportspeople with a concussion from a shock during sport practice occurring less than 72 h before inclusion were treated for 20 min at day 0 (D0) and D7 and followed up to D52. The primary outcome was the incidence of adverse device effects (ADEs). The secondary outcomes evaluated oculomotor, balance and executive function, concussion symptoms, and blood markers. The sample size was determined by the probability of observing at least one ADE, while all secondary analyses were exploratory and unadjusted for multiplicity.

**Results:**

Fifty patients were included, randomized, and treated at least once with 5 Hz (*n* = 26) or 10 Hz (*n* = 24); 49 (98.0%) of them completed the study. No difference was detected between the groups for primary or secondary outcomes. At least one ADE occurred in 38.5% (*n* = 10) [CI_95_: 20.2; 59.4] in the 5 Hz group and 41.7% (*n* = 10) [CI_95_: 22.1; 63.4] in the 10 Hz group. All ADEs were mild, non-serious (e.g., somnolence and medical device site discomfort), and resolved. Changes in several secondary outcomes were observed over the follow-up period, including within-visit changes in oculomotor measures.

**Conclusion:**

No difference in tolerability was detected between the 5 and 10 Hz modalities, although the study was neither designed nor powered to establish equivalence. Both conditions were well tolerated. In the absence of a sham comparator, secondary outcome data are hypothesis-generating and cannot be described in terms of efficacy. However, these data still provide the feasibility and variability estimates required to design an adequately powered, double-blind, sham-controlled trial in an indication that currently lacks disease-modifying treatment.

**Clinical trial registration:**

https://clinicaltrials.gov/study/NCT05647304, NCT05647304.

## Highlights

- This novel combined photonic and static magnetic field (SMF) neuromodulation device was well tolerated at both 5 and 10 Hz frequencies when used for 20-min treatment sessions in adult sportspeople with acute concussion, with no serious adverse device effects (ADEs) observed.
- No difference between the 5 and 10 Hz modalities was detected, either in tolerability or in concussion-related parameters. The study was only powered to answer the safety endpoint and therefore cannot support a claim of equivalence between the two modalities.
- Changes in oculomotor, balance, executive, and symptom measures were observed during follow-up, within a single visit. Since the trial included no sham comparator, these changes cannot be attributed to the intervention. The data do, however, establish the feasibility of recruiting and treating sportspeople within 72 h of concussion and evaluating these outcomes in that time window. They also provide the variability estimates needed to power a future sham-controlled trial.

## 1 Background

Sport-related concussion was defined by the Amsterdam 2022 International Consensus Statement on Concussion in Sport as *“a traumatic brain injury caused by a direct blow to the head, neck or body, resulting in an impulsive force being transmitted to the brain that occurs in sports and exercise-related activities, (which) initiates a neurotransmitter and metabolic cascade, with possible axonal injury, blood flow change and inflammation affecting the brain”* (1). It is characterized by an immediate, transient, and spontaneously reversible alteration in psychological and neurological functions, ranging from confusion to loss of consciousness (2). Symptoms—which include headache, vision changes, balance disturbance, somnolence, and cognitive impairment—may be immediate or evolve over minutes or hours, and usually resolve within days, but may be prolonged (1–3).

Classified as a mild traumatic brain injury, concussion was long considered benign, but it is now recognized that not all cases fully recover, with some individuals experiencing chronic sequelae lasting months or years (4, 5). Research also suggests that concussion may increase the risk of developing neurodegenerative diseases in the long term (5, 6). In sport, concussion has become a major public health concern, prompting numerous recommendations in recent years to guide safe return-to-play protocols and prevent persistent symptoms (1, 3, 7).

Current standard of care for concussion relies on symptom management; there is a lack of disease-modifying treatments (1). Considering this limitation, as well as the concussion’s burden on patients’ brains, mental health, and wellbeing (1, 8), and the associated loss of working capacity (1, 9), there is a need for immediate post-shock treatment that enables faster recovery, reduces symptoms’ duration, and potentially mitigates long-term neurological risks. In this context, neurostimulation therapies—such as transcranial magnetic stimulation, transcranial electric stimulation, and brain photobiomodulation (bPBM)—have been evaluated for traumatic brain injury, with encouraging findings to date, although further randomized clinical trials are warranted to confirm their benefits (10–14).

bPBM refers to the application of red to near-infrared (NIR) (*λ* = 600–1,100 nm) non-ionizing and non-thermal light over the brain using lasers or light-emitting diodes (LEDs) (14, 15). Discovered more than 50 years ago, photobiomodulation (PBM), previously known as low-level laser therapy, has been shown to improve tissue healing, promote cell survival, and reduce inflammation and oxidative stress in preclinical settings (16–18). Although its mechanism of action remains to be fully elucidated, bPBM is thought to rely on the conversion of light energy into metabolic energy. Photons emitted from red to NIR light would be absorbed by cytochrome *c* oxidase (CcO; complex IV of the mitochondrial electron transport chain), a chromophore and enzyme located in the inner membrane of mitochondria. Through chain reactions, PBM would increase the activity of CcO and the synthesis of adenosine triphosphate (ATP). This, in turn, would enhance mitochondrial function and initiate a cascade of cellular processes, including the induction of proteins that regulate the production of inflammatory factors, growth factors, and cytokines. Ultimately, this would result in anti-inflammatory effects, increased blood flow, neurogenesis, and synaptogenesis (15–17, 19).

The aim of this study was to investigate the safety of a novel neurostimulation medical device, the helmet RGn550 (manufactured by REGEnLIFE), which uses bPBM in association with an SMF, in treating sportspeople with acute concussion, as well as describing parameters impacted by concussion in patients under bPBM therapy.

## 2 Methods

### 2.1 Study design

The RECOVERY study was a prospective, randomized, single-blind, monocentric, pilot clinical study (NCT05647304) that compared two bPBM treatment modalities (pulsed photonic sources at the same dose but with frequencies of 5 or 10 Hz) to explore whether the two frequencies differed in tolerability and concussion-related parameters. The two pulse frequencies tested were selected within the physiological range of cortical oscillations. Pulsed tPBM has been shown to modulate neural oscillations in a frequency-dependent manner (20) and both values fall within the range in which repetitive transcranial magnetic stimulation increases neuronal activity (≥ 5 Hz) rather than decreasing it (21). No *a priori* hypothesis of superiority of one frequency over the other was pre-specified, as the comparison aimed to inform the selection of stimulation parameters for future trials. A centralized randomization sequence was created by the contract research organization (CRO) that conducted the study on behalf of the sponsors (RCTs), using SAS^®^ Viya v9.4 software, with a 1:1 allocation ratio using a block size of 2. It was stratified by gender and concussion history within 6 months prior to inclusion, as such parameters are known to influence current concussion symptoms and complications (22–24). Patients were treated according to their assigned group for 20 min during two treatment sessions: at the inclusion visit (day 0 [D0]) and 7 days later (D7). Treatment was administered by trained personnel under the supervision of a physician. Only patients were blinded to the allocated frequency. Randomization and all statistical analyses were performed by the CRO, independently from the sponsor. Patients were then followed for 52 days through 3 visits: one onsite visit at D7, one remote visit (call) at D14, and one onsite visit at D52. Other treatments involving PBM or magnetic fields, as well as benzodiazepines and antidepressants, were prohibited during the investigation to evaluate only the studied medical device.

The primary objective of the study was to evaluate the safety of bPBM therapy through the incidence of ADEs. The secondary objectives were to describe concussion symptoms, blood markers involved in concussion pathogenesis, and functions known to be impacted by concussion [balance, executive functions, and oculomotor functions, whose role in the assessment of concussion severity is the focus of growing interest (25, 26)], in patients under bPBM therapy.

The study was conducted at the AP-HP Hôpital européen Georges-Pompidou (Paris, France) between October 2022 and June 2023. It was approved by an independent Ethics Committee in France (*Comité de protection des personnes Ouest V*, 2022-A01319-34, 18 August 2022) and was conducted in accordance with the ethical principles laid down in the Declaration of Helsinki and all applicable regulatory requirements. All participants provided written informed consent.

### 2.2 Study population

Patients eligible for inclusion were adult athletes with a concussion resulting from a shock that occurred during sport practice less than 72 h before the inclusion visit, as confirmed by neurological examination via the Head Injury Assessment—Form 3 (HIA3) tool (27). Inclusions were directed by the principal investigator, who conducted them together with the co-investigator, both of whom specialize in sport-related concussion. If patients met the eligibility criteria, they would be offered the opportunity to participate in the trial. Non-inclusion criteria are mainly related to device contraindications (e.g., pregnant women, head surgery in the past 3 months, skin lesions on the head, and patients with ferromagnetic material), as well as physical, neurological, and psychiatric disorders that might interfere with study assessments (e.g., patients addicted to alcohol or drugs and patients treated with antidepressants or benzodiazepines).

### 2.3 Studied medical device

RGn550 (REGEnLIFE) is a modular helmet composed of three optical sources (red LEDs, NIR LEDs, and NIR lasers) to deliver PBM therapy, with the addition of an SMF. Each of the modules of the device contains a 5-mm-thick annular neodymium magnet generating an SMF of 280 mT. Transcranial SMF stimulation is considered to act at the synaptic level by altering membrane phospholipids and voltage-gated calcium channels, thereby modulating cortical excitability (28–30). Its intended role in this device is to modulate cortical excitability in a way that may facilitate the cellular response to PBM. The technical specifications of optical components are described in Table 1.

**Table 1:** Technical specifications of RGn550’s optical components.

Specification	Red LED	NIR LED	NIR laser diode
Total number	60	60	80
Wavelength (nm)	660	850	
Mode	Pulsed: 5 or 10 Hz depending on the group, 50% duty cycle		
Beam area (nm^2^)	929.6	94.8	102.6
Power density (irradiance max.–mW/cm^2^)	25.46	28.76	21.36
Dose (fluence max. for 20 min exposure – J/cm^2^)	15.28	17.26	12.82
Cumulated dose (for two treatments – J/cm^2^)	30.56	34.52	25.64

LED, light-emitting diode; NIR, near infrared.

Devices producing a 5 Hz frequency or a 10 Hz frequency were provided to the site. To maintain single blinding, devices were labeled with letters, without an indication of frequency. The correspondence between letters and frequency was only between REGEnLIFE and the site.

### 2.4 Outcomes and outcome measures

All outcomes were assessed by the investigators. Adverse events were collected throughout the follow-up period by the investigators: after the treatment session at D0, before and after the treatment session at D7, and finally at D52. Oculomotor and balance functions were evaluated at D0 and D7—both before and after treatment to capture any immediate effect—as well as at D52. Oculomotor function was assessed through convergence and ocular deviation. Indeed, ocular abnormalities and convergence insufficiency are emerging oculomotor-related outcomes after a sport-related concussion (31–33). These outcomes were measured through the near point of convergence (NPC) and through the Cover test and Maddox rod test for ocular deviations (34–36). Balance function, which is impacted by the following neurological impairments, was assessed through stabilometric parameters measured with KFORCE Plates (manufactured by KINVENT HELLAS) (statokinesigram area, left/right distribution, Romberg quotient, and statokinetic distribution index) (37–39). Executive function was evaluated at D0 after treatment and at D7 before treatment in all patients except four through the trail-making test parts A and B (TMT A&B), which was found to be affected by organic brain damage (40). Concussion symptoms were evaluated at D0 and D7—before treatment—as well as at D14 and D52 through the symptom evaluation part of the Sport Concussion Assessment Tool - fifth edition (SCAT5) (7). Finally, blood samples were collected at D0—before treatment—and at D52 to measure the concentration of the following blood markers: interleukin (IL)-1 receptor antagonist, IL-4, IL-6, IL-10, IL-11, and IL-13 (anti-inflammatory cytokines released as part of the neuroimmune response to concussion (41)) as well as S100 calcium-binding protein B (S100B), glial fibrillary acidic protein (GFAP), and ubiquitin C-terminal hydrolase L1 (UCH-L1) [regarded as biomarkers of acute brain injury (42)].

### 2.5 Statistical methods

All analyses were performed on patients who were treated at least once. Patients were analyzed and treated. A nominal two-sided significance level (type I error rate) was set at *α* = 0.05 for each individual test. No adjustment for multiplicity was performed. Continuous data were described by the following variables: *n* (number of patients with non-missing data), mean, standard deviation (SD), minimum, maximum, median, first quartile (*q*1), and third quartile (*q*3). Categorical data were described by the frequency and percentage of patients in each category.

For the primary endpoint, the proportion of patients with at least one ADE was calculated, along with the 95% confidence interval (CI_95_), by treatment groups and overall, using the exact Clopper–Pearson method.

For all secondary endpoints (apart from blood markers, the Romberg quotient, and the statokinetic distribution index), a mixed-effects regression model (a logistic model for categorical parameters and a linear model for continuous parameters) was used with a patient random effect. Group effects, period effects, and group-by-period interaction (for the linear model only) were added as fixed effects. Since it is a stratification variable, gender was added as a covariate in the model. Of note, concussion history within 6 months prior to inclusion, the other stratification variable, was not included as a covariate due to the low number of patients for this variable. Missing data were handled by the mixed model.

In addition, the evolution of these secondary endpoints was evaluated without distinction between treatment groups to assess overall change over time. The same mixed-effects regression model was used with the period effect and treatment group as fixed effects. As this analysis includes no sham comparator, it supports description rather than causality.

For blood markers, the Romberg quotient, and the statokinetic distribution index, only descriptive analyses were performed.

Considering a probability of ADE occurrence of 4% based on the incidence of ADEs obtained in a clinical study evaluating a previous version of the studied medical device in Alzheimer’s disease treatment (43), it was planned to include 50 patients. For such a sample size, the probability of observing at least one ADE was 0.87.

Sample size calculations and all analyses were performed using SAS^®^ Viya v9.4 software.

## 3 Results

### 3.1 Population

In total, 50 patients were included between 11 October 2022 and 02 May 2023, randomized and treated at least once with 5 Hz (*n* = 26) or 10 Hz (*n* = 24). Among them, 49 (98.0%) completed the study; 1 (2.0%) from the 5 Hz group was not included due to consent withdrawal (Figure 1). The mean ± SD follow-up duration was 1.7 ± 0.2 months.

**Figure 1:**
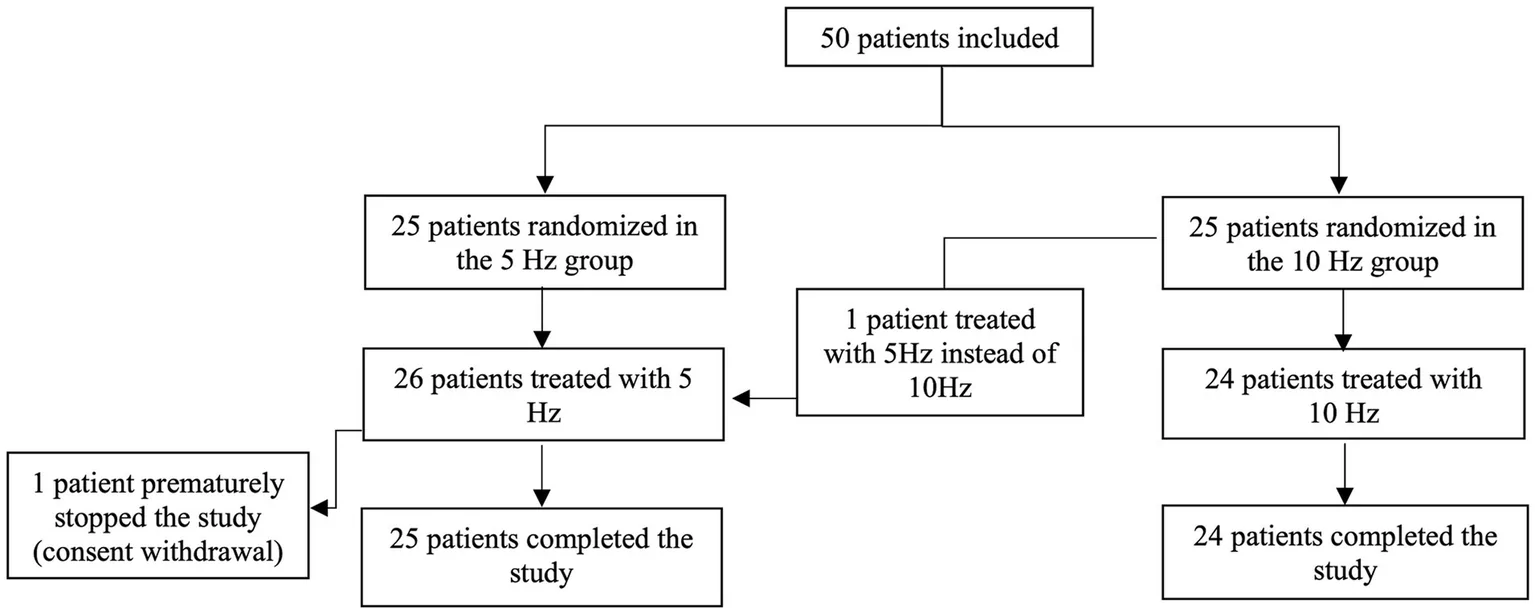
Patient flowchart.

Some deviations from the study protocol occurred during the study. TMT A&B evaluation at D7 was performed *before* treatment rather than *after* for all patients except for four. D14 visit was performed at D13 or D15 for three patients. Finally, for practical reasons related to site availability, the D52 visit was not performed on D52 but was instead conducted between D49 and D77.

Baseline characteristics of patients are described in Table 2. Overall, 88.0% of patients were male individuals, and 75.0% were aged less than 28 years. The mean time since the concussion was 2.3 ± 0.5 days, with rugby being the most practiced sport at the time of the concussion (96.0%), and the remaining being soccer players (4.0%). Almost two-thirds of patients (64.0%) already had a concussion in the past, mostly between one and three concussions (78.1%). Two patients (4.0%) had a concussion within 6 months prior to inclusion. Of note, these two patients ended up in the same group (10 Hz), despite randomization with block sizes of 2 and stratification based on concussion history within 6 months prior to inclusion, due to a data entry error in the electronic case report form.

**Table 2:** Baseline characteristics, in the overall population and per group.

Characteristic	5 Hz *N* = 26	10 Hz *N* = 24	Total *N* = 50
Gender [*n* (%)]			
Male	23 (88.5)	21 (87.5)	44 (88.0)
Female	3 (11.5)	3 (12.5)	6 (12.0)
Age (years)			
Mean (SD)	24.9 (5.2)	25.1 (5.1)	25.0 (5.1)
Median	22.9	25.1	23.5
q1; q3	21.4; 28.0	20.3; 28.5	20.8; 28.0
min; max	19.2; 40.1	19.0; 34.9	19.0; 40.1
Body mass index (kg/m^2^)			
Mean (SD)	27.7 (2.7)	27.7 (4.1)	27.7 (3.4)
Median	28.1	27.3	28.1
q1; q3	25.7; 29.2	25.1; 29.7	25.4; 29.4
min; max	21.6; 32.9	21.6; 38.6	21.6; 38.6
Time since concussion (days)			
Mean (SD)	2.4 (0.5)	2.3 (0.4)	2.3 (0.5)
Median	2.0	2.0	2.0
q1; q3	2.0; 3.0	2.0; 2.5	2.0; 3.0
min; max	2.0; 3.0	2.0; 3.0	2.0; 3.0
Practiced sport leading to concussion [*n* (%)]			
Rugby	25 (96.2)	23 (95.8)	48 (96.0)
Soccer	1 (3.8)	1 (4.2)	2 (4.0)
History of concussion [*n* (%)]	18 (69.2)	14 (58.3)	32 (64.0)
Number of past concussion(s)			
1 [*n* (%)]	6 (33.3)	4 (28.6)	10 (31.3)
2 [*n* (%)]	3 (16.7)	6 (42.9)	9 (28.1)
3 [*n* (%)]	5 (27.8)	1 (7.1)	6 (18.8)
4 [*n* (%)]	1 (5.6)	1 (7.1)	2 (6.3)
5 [*n* (%)]	0 (0.0)	2 (14.3)	2 (6.3)
6 [*n* (%)]	2 (11.1)	0 (0.0)	2 (6.3)
8 [*n* (%)]	1 (5.6)	0 (0.0)	1 (3.1)
Duration of previous concussion (days)			
Mean (SD)	10.0 (13.3)	10.9 (13.8)	10.4 (13.3)
Median	3.5	7.0	5.5
q1; q3	0.0; 15.0	3.0; 10.0	1.5; 12.5
min; max	0.0; 40.0	0.0; 49.0	0.0; 49.0
History of concussion within 6 months prior to inclusion [n (%)]	0 (0.0)	2 (8.3)	2 (4.0)

SD, standard deviation.

### 3.2 Safety

The proportion of patients who had at least one ADE was 38.5% (*n* = 10) [CI_95_: 20.2; 59.4] in the 5 Hz group and 41.7% (*n* = 10) [CI_95_: 22.1; 63.4] in the 10 Hz group. No difference was detected between the two groups in terms of safety.

Overall, 20 patients (40.0%) experienced at least one ADE during the investigation, for a total of 30 ADEs. All were mild and non-serious, none led to treatment discontinuation, and all were resolved. One event recurred in the same patient; multiple events occurring in a single patient were otherwise of different types. Of the 30 events, 18 (60%) correspond to items of the SCAT5 symptom checklist, including somnolence (15 events), photophobia, fatigue, and irritability (1 event each), and 11 (36.7%) are related to medical device site discomfort and warmth (11 patients, 22.0%, 11 ADEs) (Table 3). As the events mapped to SCAT5 items are part of the symptomatology of acute concussion itself, and as any event occurring after a session was attributed to the device in the absence of a sham control, they cannot be distinguished from the natural course of the condition and may reflect it rather than a real device effect.

**Table 3:** ADEs, in the overall population and per group.

Adverse device effect	5 Hz *N* = 26			10 Hz *N* = 24			Total *N* = 50		
	Number of ADEs	*n*	%	Number of ADEs	*n*	%	Number of ADEs	*n*	%
Any ADEs	14	10	38.5	16	10	41.7	30	20	40.0
Eye disorders	.	.	.	1	1	4.2	1	1	2.0
Photophobia	.	.	.	1	1	4.2	1	1	2.0
General disorders and administration site conditions	6	6	23.1	6	5	20.8	12	11	22.0
Fatigue	.	.	.	1	1	4.2	1	1	2.0
Medical device site discomfort	5	5	19.2	4	4	16.7	9	9	18.0
Medical device site warmth	1	1	3.8	1	1	4.2	2	2	4.0
Nervous system disorders	7	7	26.9	9	8	33.3	16	15	30.0
Hypersomnia	1	1	3.8	.	.	.	1	1	2.0
Somnolence	6	6	23.1	9	8	33.3	15	14	28.0
Psychiatric disorders	1	1	3.8	.	.	.	1	1	2.0
Irritability	1	1	3.8	.	.	.	1	1	2.0

ADE, adverse device effect.

### 3.3 Secondary endpoints related to the evolution of concussion

No between-group difference was detected for any secondary endpoints. The results below therefore describe all secondary analyses as exploratory.

Oculomotor parameters changed throughout follow-up, with the largest changes occurring right after treatment (Table 4). The adjusted mean NPC, cover test mean deviation score (considering both eyes), risk of Maddox rod test horizontal deviation, and risk of Maddox rod test vertical deviation of at least one eye were all lower at each follow-up point compared to D0 before treatment (Figure 2).

**Table 4:** Evolution of oculomotor function in the overall population.

Oculomotor parameter	D0—before treatment *N* = 50	D0—after treatment *N* = 50	D7—before treatment *N* = 49	D7—after treatment *N* = 49	D52 *N* = 49
NPC (cm)					
Mean (SD)	3.8 (4.6)	1.2 (2.1)	0.8 (1.7)	0.6 (1.4)	0.7 (1.5)
Median	3.5	0.0	0.0	0.0	0.0
q1; q3	0.0; 6.0	0.0; 2.0	0.0; 0.0	0.0; 0.0	0.0; 0.0
min; max	0.0; 20.0	0.0; 10.0	0.0; 7.0	0.0; 6.0	0.0; 6.0
Cover test mean deviation score (both eyes) [*n* (%)]					
0 (normal fixation)	7 (14.0)	20 (40.0)	20 (40.8)	30 (61.2)	29 (59.2)
1 (light restitution movement)	20 (40.0)	17 (34.0)	17 (34.7)	11 (22.4)	13 (26.5)
2 (important restitution movement)	15 (30.0)	9 (18.0)	8 (16.3)	5 (10.2)	2 (4.1)
3 (deviated eye without restitution movement)	8 (16.0)	4 (8.0)	4 (8.2)	3 (6.1)	5 (10.2)
Maddox rod test deviations [*n* (%)]					
Horizontal deviation	35 (70.0)	24 (48.0)	23 (46.9)	20 (40.8)	25 (51.0)
Vertical deviation of at least one eye	16 (32.0)	8 (16.0)	5 (10.2)	3 (6.1)	4 (8.2)

NPC, near point of convergence.

**Figure 2:**
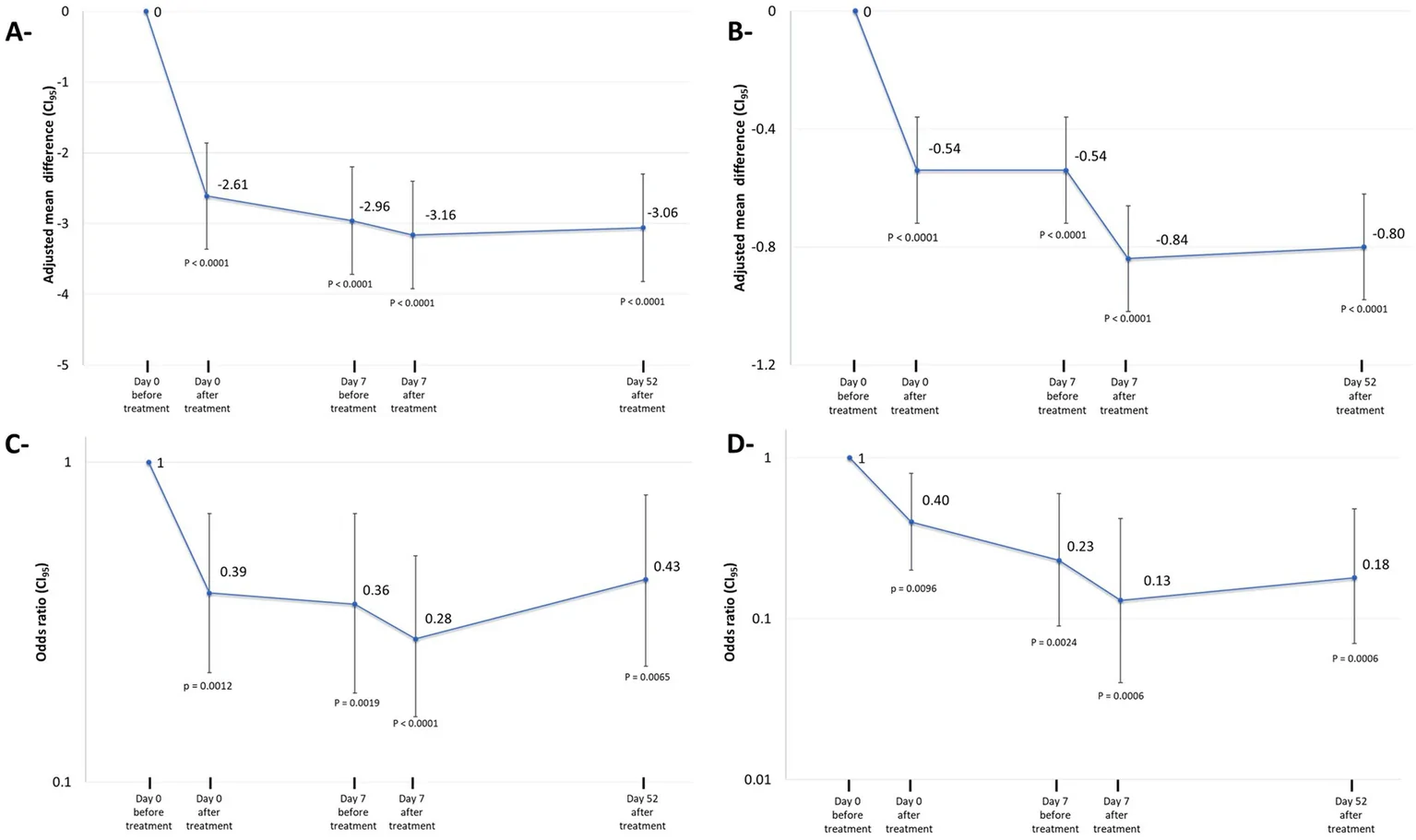
Results from linear **(A,B)** and logistic **(C,D)** mixed effect regression models—**(A)** NPC (p_visit_ < 0.0001), **(B)** Cover test mean deviation score considering both eyes (p_visit_ < 0.0001), **(C)** Maddox rod test horizontal deviation (p_visit_ = 0.0019), and **(D)** Maddox rod test vertical deviation of at least one eye (p_visit_ = 0.0095).

In particular, a decrease in mean NPC was observed after the first treatment at D0 (from 3.8 ± 4.6 to 1.2 ± 2.1 cm), with the percentage of patients with a normal NPC (i.e., NPC ≤ 5 cm) increasing from 64.0 to 98.0%. Of note, all patients with abnormal NPC before treatment except one (94.4%) returned to a normal NPC following treatment. Then, the mean NPC continued to decrease, stabilizing approximately 0.6 ± 1.4 cm or 0.7 ± 1.5 cm at both D7 after treatment and D52.

In addition, regarding the cover test, at D0, only 14.0% of patients had normal fixation (i.e., the Cover test mean deviation score of 0 in both eyes) before treatment; this proportion increased to 40.0% following treatment. It remained mostly stable at D7 before treatment (40.8%) and increased to 61.2% after treatment. At D52, this number was 59.2%.

Regarding other secondary endpoints, some parameters related to balance function (Romberg quotient and statokinetic distribution index with closed eyes; Table 5), executive function (TMT A/B—time to perform the task; Table 6) and concussion symptoms (SCAT5—total number of symptoms and symptom severity score; Table 7) decreased across follow-up, while others did not (statokinesigram area with closed eyes, difference between left and right distributions with closed eyes, TMT A/B-–number of errors).

**Table 5:** Evolution of balance function in the overall population.

Balance parameter	D0—before treatment *N* = 50	D0—after treatment *N* = 50	D7—before treatment *N* = 49	D7—after treatment *N* = 49	D52 *N* = 49
Romberg quotient					
Mean (SD)	2.1 (1.4)	1.7 (0.8)	1.8 (0.8)	1.7 (0.9)	1.7 (0.7)
Median	1.8	1.5	1.8	1.5	1.6
q1; q3	1.4; 2.3	1.1; 2.3	1.2; 2.3	1.2; 1.9	1.2; 2.0
min; max	0.7; 9.3	0.5; 3.9	0.4; 4.2	0.4; 4.8	0.6; 4.8
Statokinetic distribution index with closed eyes (mm^2^)					
Mean (SD)	91.2 (209.8)	78.9 (73.7)	65.1 (40.0)	62.6 (44.8)	58.7 (41.6)
Median	50.6	58.8	49.6	50.1	49.9
q1; q3	34.1; 77.2	45.8; 73.2	40.9; 74.5	36.9; 69.3	31.2; 62.5
min; max	23.4; 1,488.5	21.4; 364.2	20.6; 195.8	13.5; 256.7	18.4; 244.4
Missing	2	4	3	4	1
Statokinesigram area with closed eyes (mm^2^)*					
Mean (SD)	53.4 (35.2)	63.7 (54.9)	53.9 (33.5)	53.9 (37.0)	51.4 (36.4)
Median	42.5	47.0	43.5	45.0	42.0
q1; q3	28.0; 69.0	38.5; 64.0	33.5; 63.0	34.8; 57.3	28.0; 55.5
min; max	20.0; 190.0	19.0; 323.0	16.5; 175.0	13.0; 210.0	16.0; 204.0
Difference between left and right distributions with closed eyes (%)					
Mean (SD)	6.4 (4.4)	6.6 (4.6)	6.6 (4.7)	5.1 (4.4)	6.2 (4.8)
Median	6.0	6.0	6.0	4.0	5.0
q1; q3	3.0; 9.0	4.0; 10.0	3.0; 8.0	2.0; 8.0	2.0; 8.0
min; max	0.0; 19.0	0.0; 22.0	0.0; 26.0	0.0; 18.0	0.0; 18.0

*One patient had an outlier statokinesigram area value at D0 before treatment. The data of this patient at each timepoint were thus removed from this analysis (D0 before treatment: 1,374; D0 after treatment: 271; D7 before treatment: 99; D7 after treatment: 76; D52: 91 mm2). SD, standard deviation.

**Table 6:** Evolution of executive function in the overall population.

Executive function parameter	D0—before treatment *N* = 50	D7—before treatment for 45 patients and after treatment for 4 patients *N* = 49
TMT A—time to perform the task (s)		
Mean (SD)	20.1 (6.3)	17.1 (4.6)
Median	19.0	16.0
q1; q3	15.0; 24.0	14.0; 20.0
min; max	11.0; 36.0	10.0; 31.0
TMT A—number of errors		
Mean (SD)	0.0 (0.2)	0.0 (0.0)
Median	0.0	0.0
q1; q3	0.0; 0.0	0.0; 0.0
min; max	0.0; 1.0	0.0; 0.0
TMT B—time to perform the task (s)		
Mean (SD)	52.3 (23.3)	42.8 (15.3)
Median	45.5	40.0
q1; q3	38.0; 60.0	32.0; 52.0
min; max	26.0; 155.0	20.0; 106.0
TMT B—number of errors		
Mean (SD)	0.5 (0.8)	0.3 (0.6)
Median	0.0	0.0
q1; q3	0.0; 1.0	0.0; 1.0
min; max	0.0; 3.0	0.0; 2.0

**Table 7:** Evolution of concussion symptoms in the overall population.

Symptom measure	Before concussion *N* = 50	D0—before treatment *N* = 50	D7—before treatment *N* = 49	D14 *N* = 49	D52 *N* = 49
SCAT5—total number of symptoms					
Mean (SD)	0.3 (0.6)	5.0 (4.2)	1.4 (3.3)	0.8 (2.9)	0.4 (2.2)
Median	0.0	4.5	0.0	0.0	0.0
q1; q3	0.0; 1.0	1.0; 8.0	0.0; 1.0	0.0; 0.0	0.0; 0.0
min; max	0.0; 3.0	0.0; 18.0	0.0; 19.0	0.0; 19.0	0.0; 15.0
SCAT5—symptom severity score					
Mean (SD)	0.6 (1.2)	9.1 (10.6)	2.8 (8.8)	1.8 (7.9)	1.2 (6.5)
Median	0.0	6.0	0.0	0.0	0.0
q1; q3	0.0; 1.0	1.0; 12.0	0.0; 2.0	0.0; 0.0	0.0; 0.0
min; max	0.0; 6.0	0.0; 62.0	0.0; 58.0	0.0; 54.0	0.0; 45.0

No major changes were observed in IL-1 receptor antagonist, IL-4, IL-6, IL-10, S100B, or UCH-L1 concentrations. GFAP concentration was lower at D52 (median: 54.4, Q1–Q3: 37.3–76.0 pg./mL) than at D0 before treatment (median: 76.7, Q1–Q3: 54.7–117.7 pg./mL).

## 4 Discussion

The RECOVERY study aimed to investigate safety as well as describe parameters impacted by concussion in patients under bPBM therapy while comparing two pulse frequencies (5 and 10 Hz). To the best of our knowledge, this is the first clinical study to evaluate the impact of two different pulse frequencies on bPBM.

### 4.1 Interpretation

The pooled analysis includes no untreated or sham comparator and therefore does not support causal inference. These results are reported to inform the design of subsequent controlled trials.

No difference was detected between the 5 and 10 Hz groups, neither in terms of safety nor in secondary outcomes. This does not indicate that the two modalities have similar effects, since with 50 patients, only large differences were detected. The tolerability profile, exclusively mild and self-limiting events, is consistent with that reported for bPBM therapy across the literature (16, 47–49) and supports the further clinical evaluation of this device in acute concussion. The 40.0% incidence should be interpreted with the understanding that, in the absence of sham control, any event occurring after a treatment session was attributed to the device, and 60% of events corresponds to items of the SCAT5 symptom items, that is, to the natural symptomatology of acute concussion. The remaining events relate to the physical interface of the helmet rather than to the stimulation mechanism. Consistent with this interpretation, a double-blind sham-controlled trial of a previous version of the device reported adverse events in 44% of participants, with no differences detected between groups (46). Separating device effects from the natural course of acute concussion will require a sham-controlled design.

Regarding concussion evolution, all changes were observed within a single visit, before and after treatment, for all oculomotor parameters. The magnitude of this change is notable: 94.4% of participants with an abnormal NPC before treatment returned to a normal value at the same visit, and mean values remained stable thereafter. Parameters of this kind typically evolve over weeks following mild traumatic brain injury, (57–59) making these changes worth reporting and testing. Spontaneous recovery is unlikely to account for a change occurring within a single visit, but repeated testing, familiarization, and regression to the mean may operate over this timescale (33). Furthermore, since the NPC breakpoint and the Maddox rod test are not instrumented but patient-reported, expectation effects cannot be excluded. These observations show that a within-visit oculomotor signal can be captured in the acute post-injury window, and they provide effect sizes and variability estimates from which a trial can be powered. A sham-controlled design with blinded, instrumented assessment would allow this signal to be tested directly.

Changes in executive function and symptoms were in the same direction as those reported in studies evaluating bPBM therapy in concussion or traumatic brain injury (47, 54–56). As those studies were also uncontrolled, this concordance does not establish an effect but indicates that the pattern observed here is reproducible across settings and populations, strengthening the case for a controlled evaluation. Improvements in balance function after bPBM have also been reported in other pathologies, including Alzheimer’s disease (51) and Parkinson’s disease (52, 53). To our knowledge, the RECOVERY study is the first to assess oculomotor and balance function in patients with concussion receiving bPBM therapy.

Some parameters related to balance and executive functions remained stable during the study. The lack of changes in the statokinesigram area and the difference between left and right distribution with closed eyes could be related to the fact that such parameters are the result of several factors (balance, vision, occlusion, and global proprioception) and there is a latency period for them to be corrected either by the natural evolution of concussion or by the treatment’s effect. The number of errors on TMT A and B was almost zero throughout the study and therefore did not improve. Finally, the median GFAP concentration was lower at D52 than at baseline. Interpretation is limited by the sampling schedule since the baseline sampling occurred 2–3 days after the injury, beyond its acute peak, and the second sample was collected more than 7 weeks later. The observed decrease in GFAP is then consistent with the known post-injury kinetics and cannot be attributed to the treatment. The same consideration applies to the other blood markers, whose concentrations were already low at baseline: for S100B and UCH-L1, the literature confirms that they are fast-response markers, with S100B blood concentration being at its highest level a few hours post-injury and then decreasing to levels similar to the pre-injury level (54, 55) and UCH-L1 having less sustained elevations over a few days than GFAP (56). These data are nonetheless informative for the trial design, as they indicate that blood markers need to be sampled within 24 h of injury and at short intervals thereafter to contribute usefully to this indication.

### 4.2 Study limitations

The following limitations bear on the interpretation of the secondary outcomes and do not affect the primary tolerability finding, which determined the trial’s design and sample size. The main limitation is the absence of a sham group. The comparison between 5 and 10 Hz was designed to explore whether the two modalities differed, but it precludes attribution of the observed changes to the treatment rather than to spontaneous recovery, repeated testing, regression to the mean, or contextual effects. The trial was not designed as an equivalence study and the confidence intervals around the primary endpoints (CI95: 20.2–59.4 and 22.1–63.4) illustrate the limited precision achievable at this sample size. Another limitation relates to partial blinding: only patients were blinded to frequency allocation, whereas investigators and assessors knew whether an assessment was performed before or after a treatment session, although no directional hypothesis was pre-specified. Moreover, some oculomotor measures are patient-reported, meaning that expectation effects cannot be excluded for the within-visit comparisons. No adjustment for multiplicity and no endpoint hierarchy was pre-specified despite the number of statistical tests performed. Conclusions can thus only be drawn from results related to the primary endpoint. The cohort was also composed mainly of male athletes, limiting extrapolation to female athletes. This is because contact-heavy sports, which are the most common setting for sport-related concussions, are still very male-dominated. To increase the proportion of female athletes, the inclusion period should have been drastically prolonged, with a limit placed on the number of male participants. This approach aimed to address the risk of under-recruiting and failing to meet the required sample size for the study’s design. Finally, the D52 visit was performed between days 49 and 77, and no sensitivity analysis accounting for this variability was pre-specified, although it should be considered when interpreting the results from the D52 timepoint.

### 4.3 Study strength and considerations for future studies

Despite these limitations, this study provides interesting initial results on the use of bPBM therapy in acute concussion, which is an important therapeutic area still lacking disease-modifying treatment in the standard of care. Devices such as the RGn550 could offer a convenient and fast solution for the early treatment of sportspeople, should their effectiveness be established. The evaluation of this therapy in acute concussion needs to progress through randomized, double-blinded, multicentric studies versus sham, an evaluation to optimize treatment parameters (e.g., dose, wavelengths, and frequency), and an evaluation to optimize treatment protocols (e.g., duration and frequency of treatment sessions). The device can be used in double-blinded settings and has already been used in a clinical study evaluating a previous version of the studied medical device in Alzheimer’s disease treatment (46). These investigations would help establish the efficacy, safety, and optimal use of bPBM across acute concussion and a broad spectrum of sport-related brain disorders, including chronic concussion and chronic traumatic encephalopathy.

## 5 Conclusion

This randomized, single-blind, monocentric, pilot clinical trial found both pulse frequencies (5 and 10 Hz) to be well tolerated in adult sportspeople with acute concussion, with all ADEs being limited to transient, self-resolving events. No difference was detected between the two frequencies, although the trial was neither designed nor powered as an equivalence study. Changes in oculomotor function, balance, and executive function, in concussion symptoms, and in GFAP concentration were observed across follow-up, including within a single treatment visit. These analyses were exploratory and uncontrolled but helped identify candidate outcomes for controlled evaluation. This study supports the favorable tolerability profile of bPBM therapy in this population and establishes the feasibility of recruiting, treating, and assessing athletes within 72 h of concussion and provides the variability estimates required to design a double-blind, sham-controlled trial, which is a necessary next step in an indication that currently lacks disease-modifying treatment.

## 6 Eligibility criteria

### 6.1 Inclusion criteria

- Male or female individuals aged at least 18 years
- Suffering from acute concussion syndrome resulting from a shock that occurred during sport practice less than 72 h ago, as confirmed by neurological examination via the Head Injury Assessment—Form 3 (HIA3) tool
- Affiliated to French social security
- Who provided a dated and signed informed consent form.

### 6.2 Non-inclusion criteria

- Patient protected by a French legal measure (“sauvegarde de justice,” “tutelle,” or “curatelle”)
- Patient not able to express his/her consent
- Patient deprived of liberty or hospitalized without consent
- Woman who is pregnant or breastfeeding, or who plans to become pregnant or breastfeeding during the investigation, or who has the capacity to conceive but is not using a reliable contraceptive method as deemed by the investigator
- Patient living in a medical facility
- Patient who experienced surgery at the treatment application area (head) within 3 months prior to inclusion
- Patient with skin lesions on the treatment application area (head)
- Patient with a short-term life-threatening pathology (e.g., evolving cancer, non-stable heart failure, and severe hepatic, renal, or respiratory failure)
- Patient diagnosed with a heart attack within 3 months prior to inclusion
- Patient implanted with ferromagnetic material
- Patient implanted with a pacemaker
- Patient with a risk of epileptic seizure or other non-degenerative central nervous system diseases
- Patient with major physical or neurosensorial disorders that may interfere with assessments
- Patient with chronic psychosis or psychotic episodes
- Patient addicted to alcohol or drugs
- Patient treated with antidepressant or benzodiazepine
- Patient who participated in another investigation/study involving the use of an investigational medical device/drug within the 30 days prior to inclusion
- Patient not able to meet treatment sessions as deemed by the investigator
- Patient not able to complete requested investigation assessments as deemed by the investigator.

## Data Availability

The raw data supporting the conclusions of this article will be made available by the authors, without undue reservation.

## Glossary

ADE: adverse device effect
ATP: adenosine triphosphate
bPBM: brain photobiomodulation
CcO: cytochrome c oxidase
CI_95_: 95% confidence interval
CRO: contract research organization
Dx: Day x
GFAP: glial fibrillary acidic protein
HIA3: head injury assessment—form 3
IL: interleukin
LED: light-emitting diode
NIR: near infrared
NPC: near point of convergence
PBM: photobiomodulation
q1: first quartile
q3: third quartile
S100B: S100 calcium-binding protein B
SCAT5: sport concussion assessment tool—fifth edition
SD: standard deviation
SMF: static magnetic field
TMT A&B: trail making test parts A and B
UCH-L1: ubiquitin c-terminal hydrolase-L1
VOR: vestibulo-ocular reflex
